# Maintaining innovation: How to make sewer robots and innovation policy work in Barcelona

**DOI:** 10.1177/03063127231207082

**Published:** 2023-11-25

**Authors:** Carlos Cuevas-Garcia, Federica Pepponi, Sebastian M. Pfotenhauer

**Affiliations:** Technical University of Munich, Munich, Germany

**Keywords:** repair and maintenance, innovation, public procurement, co-creation, robotics

## Abstract

This article explores how innovation logics infiltrate problem and value definitions in maintenance and repair, and how innovation itself depends on considerable, often invisible care work beyond the seemingly smooth entrepreneurial narratives. We build on a growing body of work in STS that investigates the relationship between innovation and maintenance and repair. This literature argues that the obsession with innovation crowds out attention to maintenance, that innovation creates future obligations of maintenance that are often not factored into technological promises, and that ordinary maintenance and repair practices are often innovative in their own right. Empirically, we explore a case where maintenance and repair become the explicit target of high-level, high-tech innovation initiatives and how, as a result, innovation logics colonize maintenance practices. Conceptually, we explore how repair and maintenance sensitivities can be applied to innovation practices to reveal the invisible work needed to align innovation instruments with socio-material and institutional configurations. Drawing on an in-depth case study of sewer inspection robots in Barcelona, we find that attempts to innovate maintenance require a symmetric effort to maintain innovation. In our case study, innovation processes as deployed by the European Commission, research consortia, and companies required substantial repair work to function reliably in specific settings. Our study shows how divergent understandings of the public good in innovation and maintenance contexts may lead to significant tensions, and that much can be gained analytically from not treating innovation and maintenance as opposites.

On 28 March 2019, Cécile Huet, Head of the Unit for Robotics and Artificial Intelligence Innovation and Excellence of the European Commission, descended into the sewers of Barcelona. More often seen in a black suit, she was barely recognizable under the required protection glasses, helmet, and PPE overall. Unfazed by the prospect of claustrophobia, human waste, rodents, and smelly and potentially toxic gases that kept other EU officers overground, she ventured into the dark and dusty underbelly of Europe’s first ‘Capital of Innovation’ (Franganillo, 2014). The place selected for this official act was the main entrance to the *El Born Cultural and Memorial Center*, a site dedicated to the preservation of ‘urban memory and local history’ (El Born, 2021).

The reason for Huet’s unusual and demanding descent into the underground of the Catalan city was innovation. Her visit was part of the final public demonstration of two robotic platforms—one aerial, one terrestrial—designed for the inspection of sewer infrastructure in Barcelona and potentially across Europe. The robots promised to make inspection and maintenance tasks safer and more pleasant for the workers, to decrease service costs, and to help keep urban infrastructure in good condition by making it possible to carry out inspection despite sporadic threats such as toxic gas flows and crumbling walls. In the long run, the robots were expected to make a positive impact on the protection of underground water by making different materials’ decay more visible and traceable.

But Huet was there to witness more than just robots. She was equally there to witness the potential and the success of Public End-User Driven Technological Innovation (PDTI), a novel innovation policy instrument developed by the European Commission together with the robotics community. PDTI epitomized several recent trends in innovation policy, all centered on the role of the state and the public sector in innovation. These include, for example, a growing interest in ‘Public Procurement of Innovation’ (PPI), the development of innovative solutions to public problems by state entities, often articulated in the form of ‘challenges’, in ‘Co-Creation’, the bringing-together of diverse stakeholders to foster socially beneficial innovation outcomes (Lipp et al., 2023; Ruess et al., 2023), and in ‘Mission-Driven Innovation’, the orchestration of public and private sector activities around ‘missions’ to steer innovation towards publicly beneficial outcomes (Kuhlmann & Rip, 2018; Mazzucato, 2019). The particular case of Barcelona sewer robotics experiments was seen as a trial of a potential template for how the European Union could identify crucial challenges from the public sector that could be solved with the help of innovation (such as robots) in the future. By extension, this scheme also promised to boost the economic competitiveness of the firms and lead cities involved, potentially unlocking multi-billion Euro markets in Europe and around the world.

In recent years, maintenance and repair have moved towards the center of scholarly attention in STS and adjacent fields (Barnes, 2016; Denis & Pontille, 2015; Jackson & Houston, 2021; Strebel et al., 2019; Ureta, 2014). This scholarship emphasizes that the material things and infrastructures that make modern life possible, such as electricity wires, water pipes, and traffic signals, are fragile and vulnerable. They ‘work’ thanks to an array of often-invisible human labor and undervalued practices of care (Puig de la Bellacasa, 2011). Maintenance and repair scholars are often openly critical of the fact that these indispensable practices are so heavily neglected by most innovation scholars. Consequently, as Denis (2018, p. 1) observes, ‘almost every member’ of this community ‘insists to distance themselves from the innovation-centric accounts that can be found in the press [and] in academic publications’. At the same time, recent STS scholarship has also paid more explicit attention to innovation as a discourse and institutionalized set of practices (Birch, 2020; Frahm et al., 2022; Irwin et al., 2021; Pfotenhauer & Jasanoff, 2017b). This literature casts a critical light on the specific power structures, inequalities, and situated understandings of progress that today’s innovation-obsessed societies afford, and what it means if all walks of life are subjected to an ‘innovation imperative’.

In this article we make two contributions to the literature. First, empirically, we examine what happens when the supposedly distant concerns of maintenance and repair become the explicit target of innovation-centric initiatives. Second, conceptually, we explore what we can learn when we apply maintenance and repair sensitivities to innovation itself. This symmetrical approach sheds new light on how innovation imperatives can colonize extant practices of infrastructure maintenance and re-configure them in the process: Innovation can potentially help address some of the vulnerabilities and perils of maintenance work to improve conditions for workers and communities. However, innovation processes and the artifacts they create impose their own logics and forms of material standardization on the environments, thus reshaping problems and values (e.g. by prioritizing competition over complementation between different versions of maintenance). On the other hand, our case study illustrates that innovation instruments depend on (often invisible) maintenance and repair work in order to reliably perform in specific socio-material settings. In order to produce useful results, considerable local knowledge and consistent fine-tuning are needed to define needs, build coalitions, and develop acceptable technologies.

## On the relations between infrastructure, maintenance, and innovation

### Infrastructures and repair

Infrastructures have long been studied by historians, STS scholars, anthropologists, and human geographers (Anand et al., 2018; Graham & Marvin, 2001; Hughes, 1988; Star & Ruhleder, 1996). Often understood as things that enable the flow of other things such as people, water, electricity, oil, and data (Larkin, 2013; Pollozek & Passoth, 2019), infrastructures affect who has access to these services, how, where, and when (Appel et al., 2018). Infrastructures often grow incrementally, bringing together diverse materials, legal regimes and forms of expertise with different histories and often incongruent forms and functions (Edensor, 2011). As representations of modernity, they can ‘signal the desires, hopes, and aspirations of a society’ (Appel et al., 2018, p. 19) but can also perpetuate power arrangements and reproduce social inequalities through their built-in material politics (Barnes, 2016; Winner, 1980). Despite their presumed durability, however, infrastructures confront the constant threat of failure, ruin, and decay (Graham, 2010; Howe et al., 2016; Velho & Ureta, 2019). For instance, one of the most well-known arguments about infrastructures—infrastructural inversion—is that they tend to remain invisible in the background during normal operation and only ‘become visible upon breakdown’ (Star & Ruhleder, 1996, p. 116).

Building on feminist scholarship, studies of maintenance and repair foreground the vulnerability of things against the backdrop of the rationalist-functionalist gaze of the planner, as well as the ways in which infrastructures and other technologies are always imperfect (Denis & Pontille, 2015; Jackson, 2014; Puig de la Bellacasa, 2011). These authors illuminate how sociotechnical systems exist in a state in between the completely functional and the completely broken, challenging Actor-Network Theory’s notions of the ‘black-box’ and ‘immutable mobiles’ (Denis & Pontille, 2020), and addressing a gap between design and use of technology (Jackson, 2019). These studies also stress that as infrastructures grow more complex, they require more and more maintenance and repair as well as an increasingly specialized set of skills and the ‘professional vision’ of the maintenance workers (Denis & Pontille, 2015; Goodwin, 1994; Graham & Thrift, 2007). Repair work is distributed across different actors and devices (Henke, 2000). It is transformed over time, involving different techniques, materials, and instruments (Edensor, 2011), and can be constituted by different regimes that may foreground ‘stability and integrity’ or ‘functionality and flexibility’ (Denis, 2018). Moreover, it can also reconfigure gender-technology relations, as when technicians shift their attention from male to female customers to explain details about technologies’ maintainability (de Wilde, 2020). Frequently, however, these tasks, along with the people who carry them out, remain invisible and unappreciated, thus reproducing a political economy and valuation hierarchy that prefers the new and shiny over the old and mundane (Star & Ruhleder 1996). As Russell and Vinsel (2018) put it, maintenance work can be seen as ‘the labor of the oppressed themselves’ (p. 13).

While most maintenance and repair scholars prioritize local practices of material repair, Henke (2019) and Henke and Sims (2020) observe that the analysis can also be more broadly conceived to address maintenance and repair at different spatial and temporal scales, focusing on discursive forms of repair. They argue that material repair ‘almost always goes along with repairs to other forms of order’ (Henke & Sims, 2020, p. 3), prompting us to look at a broader range of activities when trying to understand infrastructures. Sociotechnical repair, they argue, can occur at the local material level but also at the systemic level.

### Innovation and maintenance

The relationship between innovation and maintenance is characterized by power asymmetries. Maintenance work is usually overshadowed by an obsession with ‘the new’ in the form of shiny and gadgety technological innovations (Edgerton, 2008; Vinsel & Russell, 2020). However, novel technologies create a need for future maintenance (de Wilde, 2020; Graham & Thrift, 2007), and maintenance and repair are the source of (often mundane) innovations produced by the skilled, creative, and sometimes improvised labor of maintenance workers and lay citizens (de Laet & Mol, 2000; Tironi, 2019; Vasilyeva, 2019). Even though maintenance and repair scholars are critical of ‘innovation-speak’, Vinsel and Russell (2020, p. 153) argue that ‘maintenance work is one area where *actual* innovation has paid off’. This area at the intersection between maintenance and innovation deserves more scholarly attention, and is the focus of this article.

In recent years, infrastructure inspection and maintenance have become focal points of innovation policy and entrepreneurial activity, with some trends such as predictive maintenance (Konux, 2022), digital twins (Anra Technologies, 2022), and autonomous or remote-controlled robots leading the pack (Flyability, 2022). Infrastructure inspection and maintenance have become the target of ambitious innovation policies at the European level. This has been driven by the argument that public sector tasks represent a huge potential market for new technologies, and by sprawling but crumbling infrastructures facing shrinking public budgets. In robotics, for example, the *Strategic Research, Innovation and Deployment Agenda 2020* (Zillner et al., 2020) established infrastructure inspection and maintenance as one of the main market segments for European robotics, alongside healthcare, agriculture, and agile manufacturing.

The politics of innovation have emerged as an active research domain in STS—including on the unequal political economy of innovation (Birch, 2020; Irani, 2019; Pfotenhauer et al., 2023), the epistemic authority over future-making (Godin, 2012; Pfotenhauer & Juhl, 2017), standardized and exclusive practices of innovation (Irwin et al., 2021; Parthasarathy, 2022), and the promissory and tech-fix logics that prominently underwrite innovation. Yet even in mainstream (pro-)innovation settings, the promises of innovation (and innovation policy) have as of late increasingly required some repair work themselves (Pfotenhauer, 2023). Frahm et al. (2022) illustrate how international organizations like the OECD and the European Commission attempt to fix the growing disillusionment with, and the problems regularly created by, innovation initiatives by enrolling society into innovation processes through the language of ‘responsibility’. They argue that these efforts to address perceived ‘democratic deficits’ in innovation are essential to the legitimacy of current liberal-democratic orders and the institutions that underwrite them. These institutional critiques resonate with the current wave of scholarship on responsible innovation (de Saille, 2015; Owen et al., 2012, 2021; Smith et al., 2021) and the turn towards public good outcomes in innovation, as present in recent calls for a ‘return of the state’ as an active driver of the innovation process (Mazzucato, 2013; Pfotenhauer & Juhl, 2017). These calls suggest that states should go beyond their passive role as funder-facilitator and take an active steering role to direct technological development, specifically with a view towards ‘societal challenges’ or ‘mission-driven innovation’ (Kuhlmann & Rip, 2018; Mazzucato, 2018; Schot & Steinmueller, 2018).

The growing emphasis on the role of the state and public good outcomes has also led to a new set of policy instruments trying to bridge the gap between tech push and need pull, and between public and private sector goals. For example, the notion of ‘Public Procurement of Innovation’ (PPI) and its sister instrument, ‘Pre-Commercial Procurement’ (PCP), have made considerable inroads in European and national public policy (Edler & Georghiou, 2007; Lember et al., 2011; Uyarra et al., 2020). In a nutshell, in PPI the state identifies public sector challenges (e.g. crumbling sewers) and elicits proposals for innovative solutions, for example, in the form of tenders or challenges. In contrast to traditional procurement that targets off-the-shelf solutions or custom-tailored devices, PPI is generally technology-agnostic and in effect tries to procure new solutions to problems. PCP, in turn, may focus on specific nascent technologies that hold promise but for which there is not (yet) a sufficient market (European Commission, 2008; Lember et al., 2014).

In this context, the intersection of innovation (policy) and maintenance is particularly interesting because the maintenance of public physical infrastructure is often delegated to the private sector through complex tendering processes and sub-contracting procedures (Charles et al., 2008; Graham & Marvin, 2001). Infrastructure maintenance is often part of larger contracts that may include multiple services such as design, construction, and management of new sections of existing infrastructure (Himmel & Siemiatycki, 2017). Sub-contracted organizations are free to decide how to find a trade-off between profit maximization and provision of maintenance service (Lozano & Sánchez-Silva, 2019). Although infrastructure procurement can lead to innovations, these are usually in construction techniques or the selection of materials that will last longer for a lower cost, rather than on maintenance practices themselves (Larsson et al., 2022). The large variation of practices within and across infrastructure sectors and the constant fluctuations in infrastructure maintenance budgeting make innovation difficult, since these do not make room for the long-term planning that innovation requires (Bouch & Roberts, 2010).

## Methods and empirical material

For this study, we drew on a number of methods and sources. Together, they provide access to complementary insights on sociotechnical repair at various scales and levels of analysis (Henke & Sims, 2020). The majority of our data is based on extended ethnographic observation from different positions and moments of the investigated project. One of the co-authors worked as a project manager in the robotics initiative from which the Barcelona sewer robotics project was coordinated, the *European Coordination Hub for Open Robotics Development* (ECHORD++), for four years (September 2014 – January 2019). During that time, she was also carrying out doctoral research in STS. All the co-authors conducted observations during public demonstrations of the robots, expert evaluation meetings, and conferences in which the projects were showcased. These events took place, among other locations, in the streets of Barcelona, laboratories, conference venues, and universities. Throughout our engagement with the project (including several months after it was officially concluded), we carried out twelve semi-structured interviews alongside numerous informal conversations with project participants, evaluators, affected workers, researchers, policy-makers, and citizens. The formal interviews lasted about one hour and were subsequently transcribed, and all participants gave their informed consent. We also conducted policy analysis of relevant materials and additional documentation of the project. Finally, we attended the launch of numerous parallel, related, or subsequent European projects on robotics for inspection and maintenance that contributed to our understanding of this emerging field of application. All the materials were coded and analysed using qualitative data analysis software.

## Sewer robots, repair, and the maintenance of innovation processes

### Taking innovation underground

On the chilly morning of 13 December, 2018, a group of maintenance workers arrived at a quiet street two blocks away from a beach in the northeast of Barcelona. Having parked their van and closed down a lane, they descended into the sewers and began to sanitize the canal environment. The reason for this particular cleaning, as one of the workers explained, was to ensure that ‘the robots won’t get damaged’. Indeed, that was an important day: Two robotics platforms were going to be thoroughly assessed as part of the final tests of the *PDTI* public procurement scheme. The teams that had developed the robots were expected to showcase the deployment of the hardware, the high-resolution image processing software, and the user interface, but there were no precise guidelines about what the evaluation would entail.

The trials went smoothly, although perhaps not as purely technological tests. In many ways, what the tests exhibited was not just the robotics platforms, but elaborate routines of care work (see also Lipp, 2022). The fairly seamless interaction between robots, developer teams, aging infrastructures, city and European officials, and maintenance workers was actually the result of three years of joint work during which certain procedures, such as robots efficiently descending through manholes with the help of maintenance workers, maintenance workers walking alongside the robots, or maintenance workers providing ahead of time the extra level of sanitation, had already been established. As became quickly evident to the observers, the workers had gotten quite used to manipulating the robots on the fly, bringing them in and out of the sewers, and performing quick routine tests and check-ups. These practices reflected the distributed character of repair work and the shifting identities of various entities involved in the maintenance process (like the sewers and the robots) from solid and to fragile (Denis & Pontille, 2017; de Wilde, 2020; Henke, 2000). This fluid routine interaction between workers, robot developers, and various material objects (including the robots themselves) was not part of the evaluation, however.

The first robotic platform consisted of a specially configured drone designed by a consortium of researchers from a local research organization, a local infrastructure construction and servicing company, and a German technical partner (henceforth ‘Team Drone’). After responding to a number of technical questions from two independent evaluators selected by the European Commission—one an experienced actor in the drone start-up sector, the other a pioneer in the nascent field of inspection and maintenance robotics—the consortium members handed the drone over to the maintenance workers. One of them lowered it through the manhole into the sewer with the help of a thin rope attached to two 3D printed hooks, where it was received by another worker.

Once in the sewer, the drone was programmed to proceed autonomously. Yet something was not working correctly. The machine kept stopping, going backwards, landing when it was not meant to, and the images transmitted to the above-ground computer was blurry. One of the technology developers asked a worker inside the sewer to clean the lenses with his finger, while telling the evaluators that, once the drone is warm, the lenses would stop suffering from condensation. While this manual fix and corollary explanation—a simultaneous material and discursive repair (Henke & Sims, 2020)—seemed to address the problem of both the drone’s progression and the reviewer’s skepticism, the drone later hit a wall and took a dive to the bottom of the sewer. The technology developer supervising the flight from his laptop said that he might have pressed a key unintentionally. After the incident, the drone could not be re-started. In response, the technology developer once again asked a worker to use his finger to check if the rotor blades could still spin smoothly. After a quick examination, the drone was brought outside for a more detailed inspection by the capable hands of two young robotics engineers. The engineers found that a piece had broken and fallen off, so a sewer worker went back down to secure its rescue. The evaluators asked if an ad-hoc test could be run outside the sewer, but in Barcelona, by law, drones cannot carry out unauthorized flights outdoors.

The second robotics platform was a six-wheel ground robot platform, slightly reminiscent of the Mars Rover, presented by a consortium that involved actors from two Spanish universities and a Portuguese robotics company (henceforth ‘Team Rover’). Their robot was much bigger and heavier than the drone, and had to be lowered into the sewer with the help of a small crane mounted to a van. In this second trial, one of the robotics specialists descended with two maintenance workers. In contrast to the drone, the six-wheeler went readily to and fro, accompanied by the workers and the researcher, for about 200 meters without apparent failure. However, due to weather conditions, the test had to end prematurely: For safety reasons, no one was allowed inside the sewers during rainy days, since water levels and flows can increase rapidly. Some workers who stayed above-ground joked that they would prefer not to have to collect both the robot *and* the researchers at the beach outlet if the water carried them away.

Later on, during lunch, the workers revealed that, jokes aside, there were sections of the sewer where people can die if they slip and fall over, where access is impossible because of an influx of gases from all four directions even after ventilation, or where accessibility has deteriorated over the years. In light of these risks, they suggested that the robots might indeed be a welcome technology. It is worthwhile to appreciate that the workers did not primarily show concerns towards potential job losses (Brynjolfsson & Mcafee, 2016; Fleming, 2019; Ford, 2015). This was partly because of the complex socio-material relationships that maintenance inevitably required even when carried out with the help of a robot, suggesting that manual labor would endure, and partly because of considerable trust built up over time towards their managers and the technology developers with whom they interacted.^1^

The exchanges during the two trials illustrate how a seemingly simple innovation challenge—applying robotics to sewer maintenance—quickly exceeded its initial framing. For one, rather than merely inspecting sewers with robots, the tests immediately entangled all actors involved in mutual maintenance, repair and care obligations centered around the robots, which underscores claims about the creation of new maintenance liabilities through technology introduction as well as ‘distributed maintenance’ and ‘relational repair’ (Denis & Pontille, 2017; de Wilde, 2020; Henke, 2000). Reminiscent of Henke’s (2000) concept of the ‘networked body’, it was clear that the robots were just one more among the multiple objects and devices that make maintenance work possible: manholes, steps, harnesses, rope, helmets and other safety equipment, brooms and buckets, and a wireless signal repeater fundamental for the robots to work. Additionally, in the case of the drone, it suggests that innovation processes are at once subject to improvised maintenance and repair efforts to shepherd the technology-in-the-making along the imagined innovation trajectory—both literally, as in ‘on track’ in the canal, as well as metaphorically, through the successful demonstration to the next development stage—and how this innovation process depended on enrolling key allies (such as the European Commission evaluators and maintenance workers) in specific roles. As Graham and Thrift (2007) note, at times it ‘becomes increasingly difficult to define what the “thing” is that is being maintained and repaired… the thing itself, or the negotiated order that surrounds it, or some “larger” entity’ (p. 4). In the following sections we explain how the local maintenance efforts required to make innovation work are complex and multiple and extend far beyond the specific test situation.

## Repairing innovation processes from above

To understand why the future of maintenance came down to a competition between two robotics platforms somewhere in the European underground, we need to shift our gaze to the European Commission and the evolution of pre-commercial procurement (PCP) instruments in Europe. PCP has been part of European Commission innovation initiatives in robotics since the early 2000s (European Commission, 2008; Lember et al., 2014). The European Commission has pushed for PCP in the hope that the purchasing power of public institutions could be channeled towards the development of new technological markets. However, by the early 2010s, PCP had still garnered little interest, with only one project proposal, in healthcare robotics, having been successfully funded in 2012, as one robotics project manager explained to us.

Prompted by conversations with the European Commission, the coordinators of the leading European flagship initiative in robotics at the time, the *European Clearing House for Open Robotics Development (ECHORD)*, decided to create a more prominent role for PCP in the future. They developed a modified version of the PCP format, which they included in the successful proposal for a follow-up European flagship initiative in robotics, later known by its acronym ECHORD++. In the process, ECHORD actors received advice from the European Commission on how previous PCP versions could be improved (or ‘repaired’, as we would argue) to overcome the unresponsive environment and to accommodate specific interests of the policy-makers. According to several interviewees, the new procurement instrument should help familiarize various actor groups (public sector organizations, companies, research labs, civil society etc.) with the idea that innovation ought to connect to public sector goals—still a relatively new innovation paradigm. ECHORD managers also proceeded to gather information on their own to understand the strengths and weaknesses of the instrument and why it had thus far largely failed to take off in Europe. As one project manager put it,

Our focus was in finding the best way of integrating public stakeholders in the technology development process …. I think the difference [with ordinary PCP] is definitely in the way the members of the core consortium are collaborating with the public authorities. Like preparing the public authority systematically in a way so that they are able to describe the challenge properly. To make them aware of what is possible with robotics and what is maybe not possible. In order to earth [sic.] their expectations. This is something where ECHORD++ and particularly urban robotics put a lot of effort in. (ECHORD++ project manager)

The resulting PCP instrument was called Public End-User Driven Technological Innovation (PDTI). It was meant to be bureaucratically simpler than its predecessors with ‘lower entry barriers’ (ECHORD++, 2019, p. 21). PDTI consisted of a quasi-standardized innovation process involving three stages, with evaluation rounds at the end of each phase that successively reduced the number of competing teams. Funded under European Commission’s Horizon2020 research and innovation funding program, ECHORD++ was coordinated by the Technical University of Munich, a major research university with a strong robotics footprint. The coordinating institution was responsible for advertising the instrument, managing the budget and overall PDTI implementation, arranging demonstrations, recruiting external expert evaluators, and keeping the European Commission abreast of developments. Two overarching areas were selected as particularly pertinent for robotics innovation: healthcare and urban services. The latter was delegated to the Institute of Robotics and Informatics at the Universitat Politècnica de Catalunya in Barcelona (UPC), an ECHORD++ consortium partner. UPC, in turn, was in charge of defining and coordinating the PDTI urban challenge—that is, identifying interesting challenges from public sector organizations that could be solved with the help of a robot, implementing a call for proposals to attract competing consortia to address the challenge, and managing the whole process.

To find the right public sector challenge for robotics, the UPC team organized a range of workshops, information days, exhibitions during science fairs across Europe, and discussions with city council representatives from various European municipalities during the 2013 Smart City Conference. They also tried to find challenges by actively reaching out to city councils all over Europe. However, these pan-European efforts yielded little success. As one manager recalled, it was extremely difficult to identify ‘the right person’ in city departments concerned with urban services to talk to about technological innovation and, more specifically, the potential role of robotics in public sector organizations (the supposed ‘Public End-Users’ of PDTI). Conversely, the UPC team also had to be careful to identify problems that would be attractive to the robotics community. Many of the challenges faced by public sector organizations did not easily lend themselves to robotics solutions. As a result, the UPC and ECHORD++ entered into a delicate match-making process where knowledge about state-of-the-art robotics and current public sector challenges in urban settings had to be considered symmetrically and translated into a joint innovation process. Concerned that they would be unable to attract mutually interesting high-quality submissions, the organizers eventually turned to local acquaintances in the public sector in Barcelona to encourage them to participate in the PDTI process, offering them guidance on how to best submit their challenges. In the end, 14 challenges were submitted by city council organizations across Europe, four of which came from Barcelona.

The challenge that was selected targeted ‘utility infrastructures and condition monitoring for sewer networks: Robots for the inspection and the clearance of the sewer network in cities’ (ECHORD++ & BCASA, 2014). It had been submitted by the Barcelona Water Authority,^2^ which became the official designated ‘Public End-User’, with assistance from a UPC professor. The Barcelona Water Authority was a newly formed organization that resulted from the merger of two entities in charge of different water services. It was founded in 2014, the same year that the PDTI started, and as a new organization, it needed to build a name for itself. The director of Barcelona Water Authority initially saw PDTI mostly as an opportunity to improve his organization’s reputation by aligning it with the city council’s goal of portraying Barcelona as Europe’s innovation capital and as a role model for other Smart Cities. As an interviewee argued:

Barcelona city council likes to be a pioneer. It likes branding Barcelona. If there’s somewhere it thinks it can participate and push for innovation, it will be there. (Project manager, Barcelona Water Authority)

Years later, in an edited volume that advertised ECHORD++, the Barcelona Water Authority stated:

Barcelona City Council, through its public body Barcelona Cicle de l’Aigua SA (BCASA), is playing an active role in guiding innovation by: identifying the public body needs; solving doubts about requirements of the service; providing existing information of the sewer network and the service offered; supporting ECHORD++ coordinators and external evaluators trough evaluations; identifying feasible locations for each testing stage; and supporting access to sewers for testing and evaluations (Varela et al., 2020).

However, sewer inspection was initially not considered a pressing challenge. Its relevance was established in conversations between the Water Authority director and the UPC professor. As this professor recalled:

[T]hen they realized the importance [of developing sewer inspection robots] because I talked with them and said take a look because you will gain here, you can gain here. If not, that would have never been a challenge (PDTI Coordinating team).

Even with this agreement, it was not immediately clear what kind of maintenance problems robots should solve for Barcelona and what the most relevant ‘challenge’ was. Barcelona’s sewer maintenance routines consisted of a patchwork of heterogeneous practices. They had been developed over many decades, shaped by the city’s centuries-old infrastructures, available maintenance knowledge and regulation, a diverse set of technologies, a steady influx of tourists and real estate investors, and labor tensions about the outsourcing of public sector services to contractor companies, among other things. To make this complex amalgamation of maintenance conditions legible to robot developers, a 30-page document called the ‘Challenge Brief’ was drafted that contained a purportedly exhaustive description of terms of the innovation competition. It stated that the sewer system of Barcelona was 1,532 km long, that more than 50 percent was 1.5 m high and therefore human-accessible, that it currently took up to six hours to inspect 1.5 km, and that the cost was about 0.75€ per lineal meter. The robots were expected to make maintenance work easier, more efficient and sustainable, with the goal to reduce costs to 0.50 € per lineal meter.

The robots had to be able to navigate autonomously and produce digital maps of the sewer, identifying manholes, home and street drainage inlets, areas of tunnel reduction or broadening, and points with sudden slope changes. Moreover, they had to be able to identify structural defects, including cracks, fractures, collapses and other points of reduced serviceability, as well as the distribution and depth of accumulated waste. This information was expected to ‘allow the sewer manager to make decisions without exposing [them] to risky locations’ (ECHORD++ & BCASA, 2014, p. 25). Additional recommended functionality included air and water monitoring and sampling systems, which would be used to inform local legislation and policy making.

Other than these specifications, the Challenge Brief was technologically agnostic. For example, there were no stated preferences for an aerial or a terrestrial solution. There were also no details about requirements on workers, how to interact with workers to make the system work, or how to decide among different options. Moreover, the teams were not expected to present an organizational plan of how they expected to collaborate with relevant stakeholders (workers, companies, the Barcelona Water Authority etc.). Yet, together with the three-stage competition structure and the budget that the PDTI had previously established, the Challenge Brief was *the* central guiding document for the innovation effort.

Six innovation consortia applied to address the urban challenge. Three were selected to participate in Phase I, one of which was eliminated after the end of that phase. The remaining consortia (Team Drone and Team Rover) were very different in their respective composition and approach, with different reasons for participating in the PDTI challenge. As noted earlier, Team Rover was formed by researchers from two Spanish universities and a Portuguese robotics company. They had been long-term collaborators and were familiar with each other's capabilities, yet neither had experience working on applications for inspection and maintenance of sewers. However, they had recently gained experience of inspection and maintenance robotics by participating in a competition in the oil and gas sector. For the Portuguese robotics company, any chance to commercialize the technology downstream would be welcome, but it was mostly seen as exploratory research to venture into a new application area. These interests matched those of the academic partners. As one of them noted:

We are very field robotics guys; actually, we define ourselves as a field robotics group. So, for us, we of course try to advance in the technology and in the state of the art but always, absolutely always considering a practical application for the thing we are researching. … And for us the use case of this problem of sewer inspection and so on we knew it was really, really into our, let us say, the thing that we can do pretty well, so we just applied for this. (Academic researcher, Team Rover)

Team Drone was more heterogeneous. The partners included a research center from Barcelona, a Spanish drone supplies company, a German company focused on sewer inspection cameras, and *Fomento de Construcciones y Contratas* (henceforth ‘Fomento’), a multinational infrastructure construction and services company who, importantly, was at that time already holding a seven-year license from the Barcelona Water Authority to conduct sewer maintenance services in the city. The fact that the Barcelona Water Authority had produced the Challenge Brief but did not carry out the inspection and maintenance work itself significantly complicated the picture of who the actual ‘Public End-User’ was, of who knew most about the sewer conditions, and of the playing field for the competition (see below).

The two proposed robotic platforms suggested different ways of conducting sewer inspection and making it part of an already complex and multiple urban assemblage that includes sewers, roads, public transport routes, and recently introduced cycling lanes (Farías & Bender, 2009). Researchers from both teams initially agreed that a drone was a riskier technology because of the humidity, poor signal, narrow space for maneuvering, and additional turbulence that the drone itself introduced to the environment. In particular, members of Team Rover who had been involved in several recent European projects on autonomous drones were convinced that the technology was not yet capable of operating inside such a ‘hostile environment’, as one researcher put it. At their best, drones might be able to explore more homogenous sewers of larger dimensions. The ground robot, on the other hand, would only be able to operate in about 50 percent of the sewer system of Barcelona because of accessibility constraints. Despite these limitations, researchers of Team Rover considered it a more robust and reliable technology for the task at hand. The researchers from the Barcelona institute in Team Drone had also considered applying with a ground robot before partnering with Fomento. However, Fomento staff had persuaded them to bet on a drone, since the company had previously used manually piloted drones in outdoor areas and were now interested in exploring the capabilities of autonomous flight. These contrasting capabilities resonate with Graham and Thrift’s (2007, p. 17) observation that maintenance and repair are ‘an ongoing process [that] can be designed in many different ways in order to produce many different outcomes’.

As illustrated during the final tests discussed above, the technological solutions differed considerably with regard to their own maintenance requirements. In the case of the drone, batteries had to be replaced or recharged approximately every 12 minutes. By contrast, the ground robot’s batteries allowed for approximately four hours of continuous inspection. On the other hand, the drone was easier to transport than the ground robot, which required a larger vehicle to fit both the robot and a crane to bring it up and down the manhole. The transport aspect is relevant because sewer maintenance operations and daily urban traffic often compete for the same above-ground space. These details illustrate Denis and Pontille’s (2015, p. 352) observation on the tacit assumptions of mundane details, such as tools running out of batteries or needing to be carried from one place to another, which ‘constitute an essential feature of the material ecology that characterizes repair and maintenance work’.

Most importantly, these details reveal different versions of maintenance that were inscribed (Akrich, 1992) in each robotic platform: The ground robot suggested extended inspection and maintenance missions that could continue uninterrupted for long periods of time. Additionally, since the team intended to add a robotic arm to collect samples relevant to the Barcelona Water Authority’s environmental responsibilities, it proposed a broader understanding of sewer maintenance (i.e. concerned about both material decay *and* sewage and sludge composition). These samples, however, were irrelevant to Fomento. The drone, by contrast, required more detailed preliminary planning in which operators would decide where exactly inspection needed robotic assistance, due to its limited battery life. These details thus shed light on the contrasting configurations of maintenance work imagined, which roles and skill requirements it projected on to the maintenance workers of the future, and which segments and elements of sewers could be serviced and maintained, how, and when.

## Repairing innovation processes from within

The previous section showed how the road to a smooth competition performance was considerably bumpier than the organizers might have expected. But why expect innovation to take place in the form of a carefully curated competition in the first place? Why put an additional set of constraints and burdens on the innovation process—a process usually imagined as flourishing from creativity driven by market opportunity and tech transfer logics (Pfotenhauer & Juhl, 2017)—and make teams compete for the ostensibly same goal, on the same timeline, and under the same set of rules?^3^ That innovation took the form of a multi-party competition was principally owed to EU regulation. To qualify as a competition and to comply with EU procurement rules, PDTI needed at least two competing consortia participating in the formal innovation process during all phases. Had either of the two remaining consortia resigned or been disqualified after any of the evaluations, the European Commission would have had to terminate the initiative altogether.

However, this requirement meant careful stage management on the part of ECHORD++ coordinators and made the innovation process vulnerable to bureaucratic disruptions and repeatedly required ad-hoc maintenance work by project managers. For example, because of administrative reasons and problems with the online grant management portal, development funding was put on hold for months, while the Phase I evaluation reports were being written. This created considerable difficulties for all teams, and the ECHORD++ project managers had to go to great lengths to convince the teams not to abandon the project lest they risk the entire 3-year process and, with it, the promise of the PDTI innovation instrument.

Several other elements made the innovation processes vulnerable to perturbations. As a case in point, about half-way during Phase I, the Barcelona Water Authority indicated to the PDTI coordinators that they preferred to see prototypes rather than mere designs and data for the robots in order to evaluate and test their performance. This ad-hoc request for a different type of demonstration was more than a superficial reporting nuisance. It effectively put the Barcelona Water Authority’s staff members in a position to impose their own understanding of what the ‘correct’ innovation process looked like—i.e. one that depended on prototypes at an early point in the development process, and where visual observation rather than technical data was considered proof of feasibility—even if they had very little prior knowledge and experience of robotics or innovation management. Their vision for the revised PDTI process suggested that innovation should proceed in a linear, physically comparable, and device-driven fashion, whereby both technology platforms iteratively improve upon prototype functionality.

These efforts in pre-structuring and intervening in the innovation process were at various times at odds with how those developing the technology envisioned their own workflows. For example, members of both teams argued that they would have preferred a more open process that did not create prototype lock-in and demonstration pressure early on, along with avoiding major alterations to their working plans three months into the already compressed project timeline. Our interviewees from the Barcelona research institute suggested that many of the performance issues they faced throughout the project had to do with the premature prototype design pressures during Phase I.

Another difficulty was the friction between the timeline of the envisioned innovation process as prescribed by the PDTI instrument owed to the Horizon2020 project scope, and the teams’ internal development routines, which (among other things) depended on their own suppliers. Team Drone suffered from delays from the company that provided the basic drone platform, and, even when delivered, the Barcelona institute researchers had to fix some production errors. The time they invested in fixing them shortened the time they had for developing the algorithms for basic flight and for carrying out tests inside the sewer. Moreover, they argued that a drone is a more complex technology than a terrestrial platform and involves more complex algorithms, which had to be carefully developed during Phase I. Yet the competitive nature and pre-determined tests of the PDTI instrument neither allowed for flexibility nor for diversified evaluation criteria that could accommodate different technology needs and timelines, which arguably led to less-than-perfect innovation outcomes and underscored the vulnerability of preconfigured innovation processes.

A final major challenge to develop these inspection and maintenance robots was the elusiveness of the notion of the Public End-User. As indicated before, the initial view of the ECHORD++ coordinators was that the Barcelona Water Authority was the designated end-user. But this public organization was not the direct beneficiary of the technology, since it usually delegated the actual maintenance work to private service providers. In practice, the Barcelona Water Authority would neither use the robots nor be affected in their own operations by their existence. There was no reason for the Barcelona Water Authority to purchase the technology if and when it would be ready. Instead, it became increasingly clear that the Barcelona Water Authority saw its own role primarily as an administrative unit that could inform future service providers of the technology’s availability and how to include it in their next contract to increase workers’ safety.

In contrast, the servicing company Fomento was a potential direct end-user, though neither a public sector organization nor an objective external observer, since it was part of Team Drone. According to some interviewees, Fomento had more extensive knowledge than the Barcelona Water Authority regarding maintenance service logistics and inspection data processing. However, due to the design of the innovation instrument, the team composition, and the EU subsidy rules that could not single out individual companies, they could not provide any input into the Challenge Brief or the structure of the PDTI instrument. At the same time, within Team Drone, partners faced informal pressure to align their work as much as possible with Fomento’s extant routines and software packages, even though these did not figure in the Challenge Brief. Fomento also imposed their own timelines and work rhythm on the other Team Drone partners, which were not always compatible with the PDTI timeline. As a result, partners occasionally felt as if they were working for two different clients (Fomento and the Barcelona Water Authority), having to comply with different sets of formal and informal guidelines and specifications.

## Late repair and disrepair

By the end of the project, it became evident that neither of the two robotics solutions would be picked up by the public body as envisioned by public procurement of innovation logic. During the final evaluation, two independent evaluators selected by the European Commission led a discussion about what the Barcelona Water Authority and the ECHORD++ coordinators could do to help the teams that had invested several years of work bring their technologies closer to the market. Their suggestions included, first, encouraging the teams to join and combine their technologies—notwithstanding their vastly different underlying technological premises, social requirements and corollary understandings of sewer maintenance practices—and to join efforts to build and shape the hitherto non-existent market of sewer inspection robots. This suggestion reflected the European Commission’s overarching interest in creating markets for European robots. It saw Barcelona’s unique maintenance needs as a mere stand-in for imagined universal challenges for cities around the globe.

Their second suggestion was for the ECHORD++ managers to organize a meeting and learn about the interests and expectations of all the stakeholders involved, to better understand their plans and possible opportunities, and to gauge what steps could be taken to make use of the PDTI results. In an ironic turn, the 2.5 year process had arrived at a point where all actors realized that they needed to better unpack the problematic category of the ‘Public End-User’.

As a third suggestion, one evaluator argued that, as cities across Europe grow, sewage infrastructures will decay more rapidly, and he envisioned that EU-wide regulations might soon restrict human entry into these dangerous underground spaces. Against this background, he encouraged the Barcelona Water Authority to lobby for the technologies among their water management networks across Europe with the aim of raising additional funds for further research and development. While a combination of the second and third suggestions was considered optimal by the two evaluators, it was quickly discarded by staff from the Barcelona Water Authority on grounds that they could not afford the financial and human resources that the third option required.

In the end, a mix of the first and second suggestion was followed. An extraordinary all-stakeholder meeting was held on 19 January 2019, during which all the PDTI participants were present. Researchers from the Drone and Rover Teams confirmed their interest in continuing work on inspection and maintenance applications. However, representatives of Fomento and the Barcelona Water Authority affirmed that neither of them was in a position to become new sponsors of the technology. Fomento argued that even though the technologies had advantages, neither was a feasible or profitable option yet. It added, to the surprise of many, that reducing costs for inspection services had never been a big incentive for the company to begin with, because inspection work only accounts for 10% of its human resource expenses on sewer maintenance. The vast majority, 180 out of 200 people, worked on actual repair.

The Barcelona Water Authority, in turn, argued that the political implications of financially supporting a technology that might be perceived as taking away workers’ jobs was more concerning than the operational costs per sewer mile. One of the evaluators responded that it would be morally wrong to keep sending people into the sewer if there were other possibilities, and that Barcelona would set a good example to other cities. But for the Barcelona Water Authority—and by extension, the City Council—human workers were, in the end, also voters. This line of argumentation suggests ambiguities about who the beneficiaries of the innovation were, as well as complex dilemmas involved in how to define what ‘public good’ meant from the perspective of a public authority.

By the conclusion of the all-stakeholder meeting, there were no concrete plans for the sewer inspection robots. Participants were acutely (and perhaps painfully) aware of the swath of open issues, limitations, and problems. The technical consortium partners lamented that the European Commission had no mechanisms in place to continue supporting research and innovation projects that had successfully jumped through every hoop put before them. The ECHORD++ coordinators bemoaned that the public entity was not legally bound to purchase either technology by the end of the project. The Barcelona Water Authority was wary of possible political fallouts.

Three years later, the technical partners of both teams have moved on to work on projects in other areas of application, such as energy and railway infrastructure inspection. The Barcelona Water Authority continued supporting innovation projects addressing daily challenges other than sewer inspection. The European Commission has funded many other projects on inspection and maintenance robotics. However, to the best of our knowledge, the PDTI instrument and other pre-commercial procurement formats, lacking continued maintenance and care beyond the project period, have not made significant inroads into European robotics innovation.

## Conclusion

The story of robotics innovation in the Barcelona sewers sheds new light on the variegated relationships between infrastructure maintenance and innovation. Bringing together two bodies of STS literature that are often treated as separate, if not antithetical—studies of maintenance and repair and of innovation —our article explores what analytic purchase can be gained by studying these activities in tandem, where maintenance itself becomes the target of concerted innovation efforts and where, conversely, maintenance and repair sensitivities can help us understand the fragility and work of innovation.

Our research shows that maintenance practices today are shaped not just by the material environments we build, the socio-material practices they afford, and the institutional frameworks we construct around them. They are also, and perhaps increasingly, shaped by innovation pressures and logics. These include the devices and corollary use routines through which we envision the future of cities and maintenance work, and how we structure the processes through which these novel devices and routines get produced. This is evidently true for past technologies that are employed today to conduct maintenance work. But it might be more true today in an era when innovation has become a goal in itself, with increasingly standardized understandings of how innovation ought to operate and tied to specific capitalist logics of valorization (Pfotenhauer et al., 2021; Pfotenhauer & Jasanoff, 2017a).

Taking the political economy of innovation and its structuring effects seriously can thus help us to better understand which extant socio-material assemblages are being challenged and displaced in the name of innovation, and how the benefits, risks, and burdens of public sector services are accordingly redistributed. It may reveal the hidden costs of innovation: the erasure of existing ways of working and living, the closure of alternative pathways to improving socially relevant work, or (at the most basic level) who and what is maintained. As we have seen, the structure of the PDTI innovation process put two different visions of inspection and maintenance in competition with each other, each with its own built-in assumptions about which parts of the sewers could be serviced, at which pace, and with what kind of additional human labor. As we noted before, this format discouraged possible efforts to combine both approaches (e.g. using the robust ground robot and its long battery life for some sections of the sewer and the more versatile but shorter-lived drone for others), which ended up being one of the (unheeded) recommendations of the external evaluators at the end of the process.

Second, our case study shows how innovation processes require substantial additional care and maintenance work, including closely interrelated forms of material and institutional/discursive repair (Henke & Sims, 2020). At no point was the introduction of robots to the Barcelona sewers a self-sustaining, smooth, or purely inventive process where technologies, market opportunities, and genius magically connected. Instead, it required labor and support from a range of actors and devices, from the introduction of novel funding schemes by the European Commission to further public interest dimensions and the joint definition of sewer target specifications across multiple stakeholders, to the management of a multi-stage competitive process based on iterative evaluations and assistance provided to robots by human workers during official tests, all the way to the post-hoc stocktaking of the misconceptions about the process itself. What is more, for the PDTI instrument to function, many of its formal features had to be re-interpreted, challenged, and adjusted along the way according to the situated needs, assumptions, possibilities, preferences, and extant routines of the participants. The degree of maintenance and care work observed may be particularly high for the curated, standardized PDTI innovation instrument constructed by the ECHORD++ consortium to be in compliance with both European research funding and procurement rules. But it is easy to see how such maintenance logics also apply to other, less formally structured innovation pathways. Many of the steps considered essential to technological entrepreneurship today, like tech transfer arrangements, business plan competitions, or fundraising rounds, are increasingly standardized and require quasi-infrastructural maintenance work to function.

More broadly, innovation success also depended on continuous and multilayered efforts of plausibilization. Roboticists and EU officials had to make local actors (like city administrators) *see* the benefits of developing robots for their sewers (Denis & Pontille, 2022), for example by enrolling local researchers or companies, referring to social welfare concerns and labor relations with maintenance workers, or catering to Barcelona’s aspiration as an ‘innovation capital’ (Akrich et al., 2002). This creation and maintenance of narrative plausibility through positioning work within established broader imaginaries resonates with Hilgartner’s (2015) notion of ‘vanguard visions’ and Pfotenhauer et al.’s (2023) analysis of efforts to legitimize innovation as an extension of existing social orders within regional innovation cultures. To that end, our case reveals an interesting dynamic of subsidiarity and delegation evident in the conflicting visions of innovation held at different levels of governance, including divergence with regard to innovation’s beneficiaries, purposes, and processes. In developing the PDTI instrument, the European Commission aimed to specifically address local societal challenges, whereas ‘local societal’ was understood as ‘proposed by a public sector entity that knows the problems of its citizens’. The Commission enacted this vision by implementing a standardized, competition-based, somewhat linear, multi-stage innovation instrument with the help of robotics experts that drew on pre-configured notions of ‘end-user’ participation, relevance, and the public. At the same time, these local configurations were assumed to be representative for all of Europe and its maintenance problems, and both the process and outcome envisioned as scalable far beyond Barcelona (Pfotenhauer et al., 2021). That is, from the European Commission’s perspective, solving local challenges was principally a passage point for creating a European market for sewer inspection robots.

This raises a number of interesting questions, including who gets to articulate innovation needs (for example, in the form of ‘challenges’ of infrastructural maintenance) and how can standardized innovation instruments be brought into conversation with locally specific problems and contexts such as maintenance and repair. These diverging understandings of where, what, how and for/with whom to innovate that are held at different levels of governance within Europe call attention to what Jasanoff (2013) calls ‘epistemic subsidiarity’—shorthand for the argument that in an era of transnationalism, the right of communities to govern themselves at the lowest level is bound up with a corollary right of these communities to know in the way they want through ‘legitimate local and national preferences for institutionalized modes of public reasoning’ (p. 136). One could argue that meaningful self-governance in an era of innovation should entail the right to define problems, processes, and criteria of innovation at the lowest feasible level, which stands in contrast to globalizing innovation dynamics (Pfotenhauer & Jasanoff, 2017a).

In this context of fragmentation and overflow, it is important to note that maintenance and innovation may both aim to serve the public good, but do so in quite different ways. Sewer maintenance is part of the vast underbelly of invisible infrastructures of urban life and the range of public services typically provided by municipalities. Their situated character is shaped by the idiosyncratic sewer architectures grown over decades or even centuries and the socio-material settlements and practices—including labor relationships, urban planning and sprawl, health and hygiene standards, practices of outsourcing public services to private contractors, etc.—that have emerged around them. In the Global North, the availability of such public services is often assumed and its absence would mean a severe violation of welfare standards. Innovation logics, in contrast, typically take private—rather than public—good aspects as a starting point, where individual innovator-entrepreneurs may reap disproportional returns from inventive activity if properly coupled with market mechanisms.

In public good-driven approaches to innovation such as PCP, these are typically envisioned as a tandem of solutions to concrete public problems and economic benefits of successful innovative businesses for a region. The public benefits are typically not assumed or present, but promised and to be realized in the future, which in turn requires some plausibilization efforts to ensure that these imagined societies and sociotechnical changes are indeed desirable for the population. These different visions of the public good inherent in innovation and maintenance logics may easily be in conflict with one another. In our case study, there was limited agreement on the right trade-off between workers’ occupational health and safety on the one hand and their future employment security on the other. There was also limited consensus on which sections of the sewer system required robot support the most, leading to two starkly different proposed platforms.

Finally, our study speaks to an interesting policy gap between public policies trying to support innovation or maintenance, respectively. On the one hand, we have seen how innovation logics, pressures and policies are creeping into other domains, with ambivalent effects. This is consistent with the observation by Pfotenhauer et al. (2019) that it is increasingly hard to frame public policy problems in ways that do not suggest innovation as part of the solution. For example, in wrestling with the prospect of a dead-end in the aftermath to the PDTI competition, it was suggested that policy responses to urban sprawl and infrastructural decay could be framed as an innovation opportunity that could further EU ambitions to establish and dominate a global market of maintenance robotics. On the other hand, innovation and maintenance logics might not gel as easily as actors might initially assume. The ECHORD++ organizing team had difficulties finding both the right urban challenges for municipal administration *and* the right interlocutors to try out innovative approaches. One may wonder whether municipalities should anticipate these proliferating innovation logics better as a way of capturing emergent opportunities and resources. At the same time, allowing innovation logics to penetrate other social domains might reproduce the starkly unequal geography of innovation into a corollary geography of (urban) public services where welfare state mechanisms become subordinated to private sector visions of how to foster the common good. Understanding which parts of public sector reform decisions are driven by innovation imperatives might clarify where certain rationales are coming from and how to construct credible alternative pathways where needed.

## Notes

1. Although in this particular case the workers welcomed the technologies, we believe that any maintenance innovation project should include clear procedures to address concerns of labor automation. These should be guided by inclusive, informed, and empowered decision-making by a variety of actors (Chilvers et al., 2018; Stirling, 2008; Wynne, 2006).

2. The actual name of the organization is *Barcelona Cicle de l’Aigua SA* (BCASA). However, in this paper we omit this and some other acronyms and opt for names that make our narrative easier to follow.

3. It is also worth noting that the PDTI rules as developed by ECHORD++ required not just competition among teams, but also that all teams be ‘international’. This requirement speaks to the fact that ECHORD++ was funded under the Horizon2020 framework program, which explicitly aims to support European collaborative research and innovation that goes beyond what could be funded by national research agencies. In follow-up work, we plan to investigate what exactly makes the ECHORD++ robots ‘European’. For now, we content ourselves with noting that the international collaboration requirement put additional constraints on who was able to respond to the challenges, what kind of maintenance visions could be represented, and how national innovation was deemed deficient from this particular PCP perspective.
